# Th1-Th17 Cells Contribute to the Development of Uropathogenic *Escherichia coli*-Induced Chronic Pelvic Pain

**DOI:** 10.1371/journal.pone.0060987

**Published:** 2013-04-05

**Authors:** Marsha L. Quick, Larry Wong, Soumi Mukherjee, Joseph D. Done, Anthony J. Schaeffer, Praveen Thumbikat

**Affiliations:** 1 Department of Urology, Northwestern University Feinberg School of Medicine, Chicago, Illinois, United States of America; 2 Department of Pathology, Northwestern University Feinberg School of Medicine, Chicago, Illinois, United States of America; University of São Paulo, Brazil

## Abstract

The etiology of chronic prostatitis/chronic pelvic pain syndrome in men is unknown but may involve microbes and autoimmune mechanisms. We developed an infection model of chronic pelvic pain in NOD/ShiLtJ (NOD) mice with a clinical *Escherichia coli* isolate (CP-1) from a patient with chronic pelvic pain. We investigated pain mechanisms in NOD mice and compared it to C57BL/6 (B6) mice, a strain resistant to CP-1-induced pain. Adoptive transfer of CD4+ T cells, but not serum, from CP-1-infected NOD mice was sufficient to induce chronic pelvic pain. CD4+ T cells in CP-1-infected NOD mice expressed IFN-γ and IL-17A but not IL-4, consistent with a Th1/Th17 immune signature. Adoptive transfer of ex-vivo expanded IFN-γ or IL-17A-expressing cells was sufficient to induce pelvic pain in naïve NOD recipients. Pelvic pain was not abolished in NOD-IFN-γ-KO mice but was associated with an enhanced IL-17A immune response to CP1 infection. These findings demonstrate a novel role for Th1 and Th17-mediated adaptive immune mechanisms in chronic pelvic pain.

## Introduction

Prostatitis is the most common urologic diagnosis in men younger than 50 years and the third most common urologic diagnosis in men over 50 years [1]. Category III prostatitis or chronic pelvic pain syndrome (CPPS) is the most common prostatitis observed in medical practice, representing 90% of chronic prostatitis and is a poorly understood entity characterized by pelvic or perineal pain, irritative voiding symptoms and sexual dysfunction [2]. A microbial etiology for CP/CPPS has long been postulated [3]–[7] but a cause and effect relationship has been difficult to demonstrate due to the chronic nature of the syndrome, the often-delayed diagnosis and the confounding presence of uropathogens in prostates of 5% of healthy men and those with CP/CPPS [3]. We recently isolated an *E. coli* strain named CP-1 from the prostate of a man with chronic pelvic pain who did not show a history or concurrent presence of acute or chronic bacterial cystitis [8]. CP-1 was characterized in an animal model as being capable of inducing chronic pelvic pain that persisted beyond the presence of bacteria in the prostate. The pelvic pain response required an amenable host environment, the non-obese diabetic (NOD/ShiLtJ) mouse. In contrast, the inflammatory response following prostate infection was observed in both NOD/ShiLtJ (NOD) and C57BL/6 (B6) mice. Given the well-known predisposition of NOD mice for autoimmunity [9] we hypothesized that CP-1-induced chronic pelvic pain in the NOD mouse was mediated by autoimmune mechanisms. We therefore examined the immune response to CP-1 infection in the pain permissive NOD mouse strain and compared the response to the chronic pelvic pain resistant B6 strain. Our results show that chronic pelvic pain in the NOD mouse is associated with autoimmune mechanisms that are mediated by IL-17A and IFN-γ-secreting CD4+ T cells. Furthermore, the immune response exhibits host and pathogen specificity that closely mimic the pelvic pain response.

## Results

### CP1 infection elicits chronic inflammation, chemokine upregulation and lymphoid enlargement

**Figure 1:**
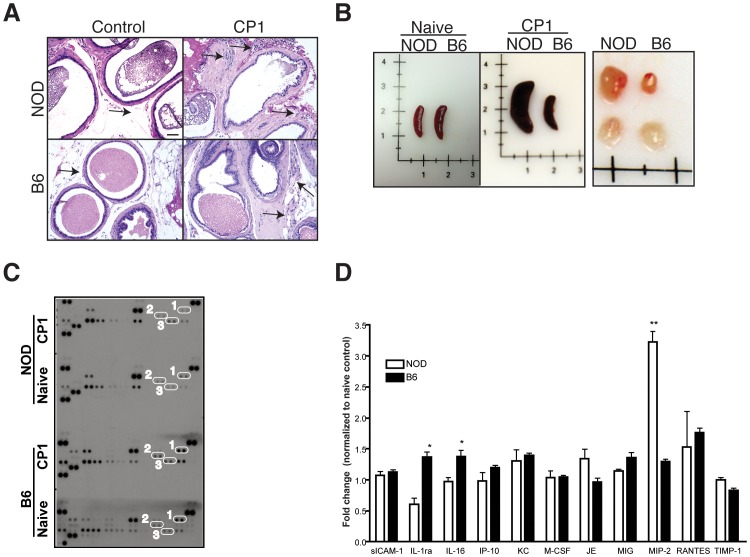
CP1 infection in NOD and B6 mice elicit differential immune responses. (A) Representative histopathology of the prostate of NOD and B6 mice 28 days after CP1 infection or no infection (controls). Arrows represent areas of leukocytic infiltrate in the prostate. Bar, 50 µM. (B) Representative images of the spleen and lumbar lymph node in naïve or CP1 infected NOD and B6 mice 28 days following infection. Lymph nodes from infected mice are displayed in the upper row. (C and D) Profile of chemokines in prostates of NOD and B6 mice 28 days following CP1 infection. Chemokine levels were normalized to naïve NOD and B6 prostates (C) and chemokines differentially expressed between NOD and B6 mice were expressed as relative fold change (D). Representative image of the immunoblot (C) indicates elevation in IL-1ra and IL-16 (1 and 2) in B6 mice and MIP-2 (3) in NOD mice. The experiment was performed with pooled prostates (n = 5/group) and repeated twice. Statistical significance is indicated at * p<0.05.

We have previously demonstrated that both NOD and B6 mice show early prostatic inflammation in response to CP1 infection, but the chronic pelvic pain response is seen only in the NOD strain [8]. We therefore hypothesized that differences in the level of chronic inflammation or changes in cytokine/chemokine profiles in the prostate may mediate the variability in response between the two mouse strains. At 30 days following CP1 infection, H&E sections of the prostates of B6 and NOD mice showed similar levels of chronic inflammation, marked by leukocytic infiltrates in the periglandular and stromal areas of the prostate lobes (Fig. 1A). We next examined the chemokine profile in the prostates of infected NOD and B6 mice (pooled prostates, five per group). Antibody arrays detected a number of cytokines that were similarly elevated in both mouse strains when compared to their respective naive controls (Fig. 1C). Significant differences between B6 and NOD mice were only observed in levels of IL-1ra and IL-16, observed to be elevated in B6 mice, and MIP-2 differentially elevated in NOD mice (Fig. 1D). In contrast, on examination of the spleen and lymph nodes of infected NOD and B6 mice at 30 days, marked enlargement of spleens and lumbar lymph nodes were observed only in the NOD strain and not in the B6 strain when compared to their respective naïve controls (Fig. 1B). Thus, NOD mice demonstrate chronic inflammation, chemokine upregulation and marked enlargement of secondary lymphoid organs in response to CP1 infection.

### Adoptive transfer of immune T cells transfers pelvic pain to naïve NOD mice

**Figure 2:**
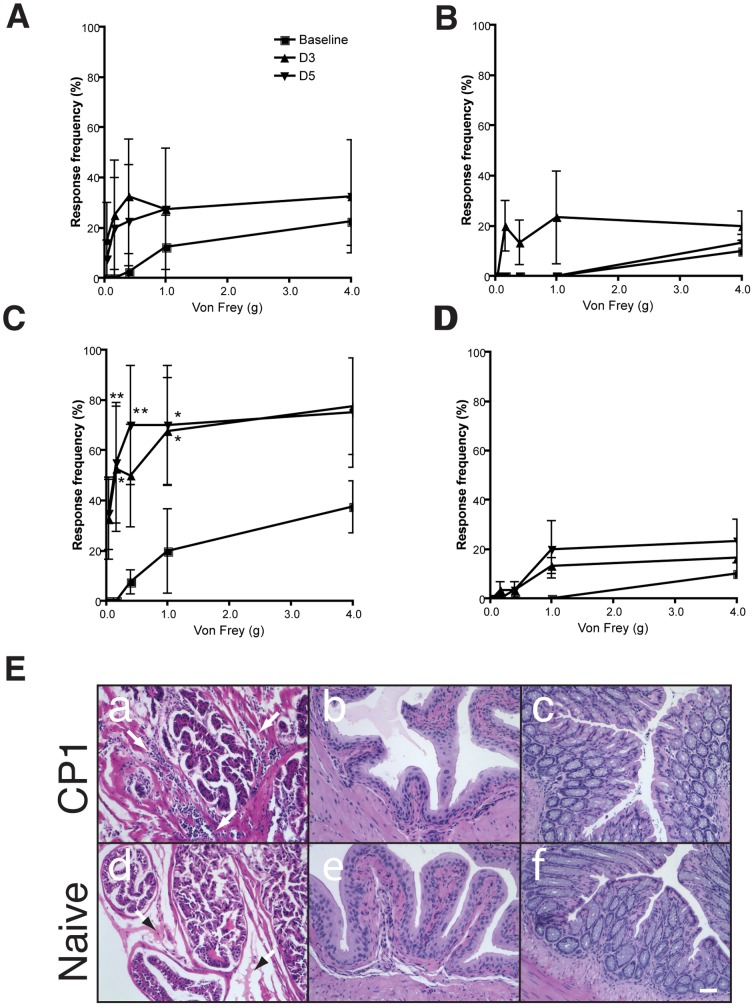
Adoptive transfer of T cells mediate pelvic pain in NOD mice. (A and B) Naive NOD mice (n = 5) were injected with 100 µl of serum from CP-1 infected (28 days) (A) or naïve (B) NOD mice and referred visceral hyperalgesia in NOD mice was measured as responses to mechanical stimulation of the pelvic region using von Frey filaments of 5 calibrated forces. Data is shown as the mean percentage of positive response ± SEM before instillation of serum or pan T Cells (baseline) and at days 3 and 5 following injection. (C and D) Naïve NOD mice (n = 5) were injected with pan T cells isolated from CP-1 infected (C) or naïve (D) NOD mouse spleen and lymph nodes and responses to mechanical stimulation of the pelvic region was measured as before. (E) All experiments were repeated at least two times and significant increase in response frequency is represented by * p<0.05 and **p<0.001. The symbol key shown in panel A applies to panels B, C and D. (E) Representative histopathology of corresponding prostate (panels a and d), bladder (b and e), and colon (c and f) of naïve or CP-1-exposed pan T cell recipients. Arrowheads in a and d indicate comparison of leukocytic infiltrates. Scale bar, 50 µM.

We sought to identify the nature of the immune response to CP-1 infection that contributes to chronic pain in NOD mice. To study this, we infected donor NOD mice with CP-1, isolated serum and T cells (Pan T) and transferred them into naïve NOD mice (five per group) that were unexposed to CP-1. Recipient mice were tested at baseline and over time for the development of suprapubic tactile allodynia, a consequence of referred hyperalgesia and a characteristic of visceral pain [10]–[12]. While tactile allodynia was not changed in the group administered immune serum or naïve serum (Fig. 2A and B), significant elevations were observed at three and five days post-transfer in the group given T cells from CP-1 exposed donor mice (Fig. 2C). In contrast, mice given naïve T cells did not show any significant elevation (Fig. 2D). On histological examination of the tissues, moderate inflammation characterized by leukocyte infiltration was noted in the prostates of CP-1 T cell recipients but not naïve T cell recipients (Fig. 2E compare panels a and d). Inflammation appeared to be specific to the prostate because no significant inflammatory infiltrates were noted in corresponding bladder or intestinal tissue sections (Fig. 2E, panels b, c, e and f). These suggest that chronic pelvic pain in mice is T cell mediated but is not dependent on a humoral response.

### CP-1 experienced CD4+ T cells transfer pelvic pain to naïve NOD mice and proliferate *ex vivo* in response to prostate antigens

**Figure 3:**
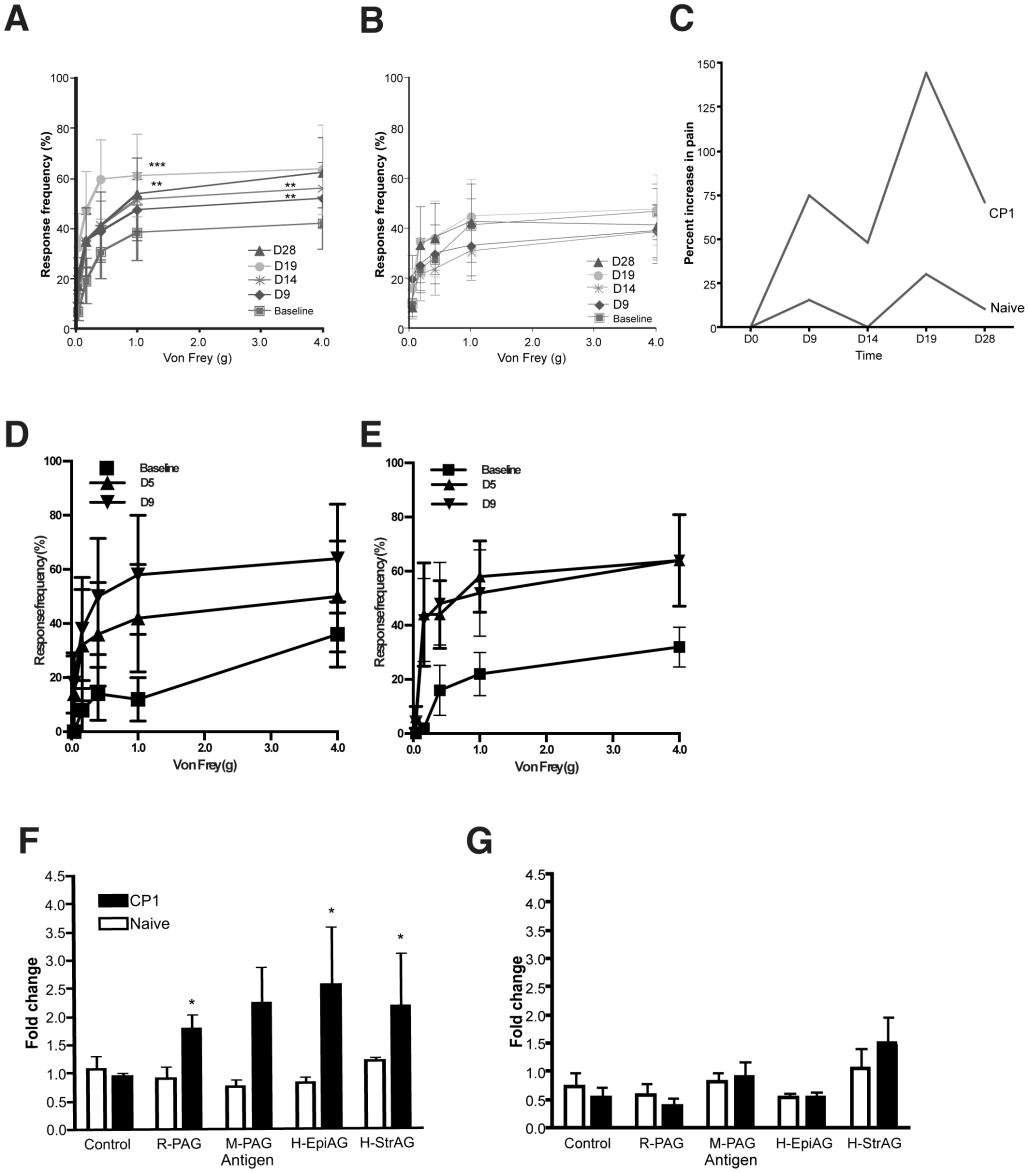
Adoptive transfer of CD4+ T cells mediate pelvic pain in NOD mice (A–E) Naive NOD mice (n = 5) were injected with 4×10^6^ CD4+ or CD8+ T cells from CP-1 infected (A and D) or naïve (B and E) NOD mice and referred visceral hyperalgesia in NOD mice was measured as responses to mechanical stimulation of the pelvic region using von Frey filaments of 5 calibrated forces. Data is shown as the mean percentage of positive response (+/−) SEM before transfer of T cells (baseline) and at PID 9, 14, 19 and 28 (A and B) or PID 5 and 9 (D and E). (C) Percent response in NOD mice injected with CD4+ T cells from CP-1 infected and naïve NOD mice monitored at day 0, 9, 14, 19 and 28 (n = 5). F) and G) CD4+ T cells from CP1 infected NOD mice show proliferative response to prostate antigens. Irradiated splenocytes from naïve NOD mice or naïve B6 mice were used to present 2 µg/ml human, mouse and rat prostate antigen to CD4+ T cells isolated from naïve or CP1 infected NOD (F) and B6 (G) mice. The proliferative response was measured as absolute cell counts per 100 µl on day 3 and expressed as fold change in cell numbers compared to day 1. Experiments were repeated independently twice and statistical significance is shown at *p<0.05.

We next examined CD4+ and CD8+ T cell subsets for their role in the mediation of chronic pelvic pain. We adoptively transferred naive or CP-1 experienced donor CD4+ and CD8+ T cells into naïve NOD recipients (five per group). In following pelvic pain behavior over 28 days, pain behavior was observed to be significantly elevated at day 28 in CP-1 CD4+ T cell recipients but not in control CD4+ T cell recipients (Fig. 3 A&B) suggesting that the CP-1 CD4+ T cell transfer was sufficient to establish and sustain a chronic pain state in recipient mice (Fig. 3C). In contrast to the specificity of the CD4+ T cell adoptive transfers, CD8+ T cell transfers from naïve (Fig. 3D) or CP-1-infected mice (Fig. 3E) were associated with pain responses that were elevated, but not significantly different from each other suggesting non-specific mechanisms. These results suggest a critical role for CP-1 induced CD4+ T cells in mediating chronic pelvic pain in NOD mice.

We examined the ability of CD4+ T cells from CP1 infected NOD and B6 mice as well as respective naïve controls to undergo proliferation in response to prostate antigens from mouse, rat and human prostates. Irradiated naïve splenocytes from NOD or B6 mice were incubated with human prostate epithelial or stromal cell antigens, mouse prostate antigens or rat prostate antigens and used as antigen-presenting cells with CD4+ T cells from NOD or B6 mice (3 per group) infected with CP1 for 28 days. At days 3 following the start of antigenic stimulation, CP1 experienced CD4+ T cells from NOD mice showed a significant increase in cell proliferation in response to human prostate epithelial antigen (H-EpiAG), human prostate stromal antigen (H-StrAG) as well as rat and mouse prostate antigens but not to controls (Fig. 3F). In contrast CD4+ T cells from naïve NOD mice (Fig. 3F) or CP1-infected B6 mice (Fig. 3G) did not show any significant increase in T cell proliferation. These results suggest that CP1 infection in the NOD mouse primes CD4+ T cells to undergo proliferation in response to prostatic antigens.

### CD4+ T cells express IFN-γ and IL-17A in a host and pathogen-specific fashion

**Figure 4:**
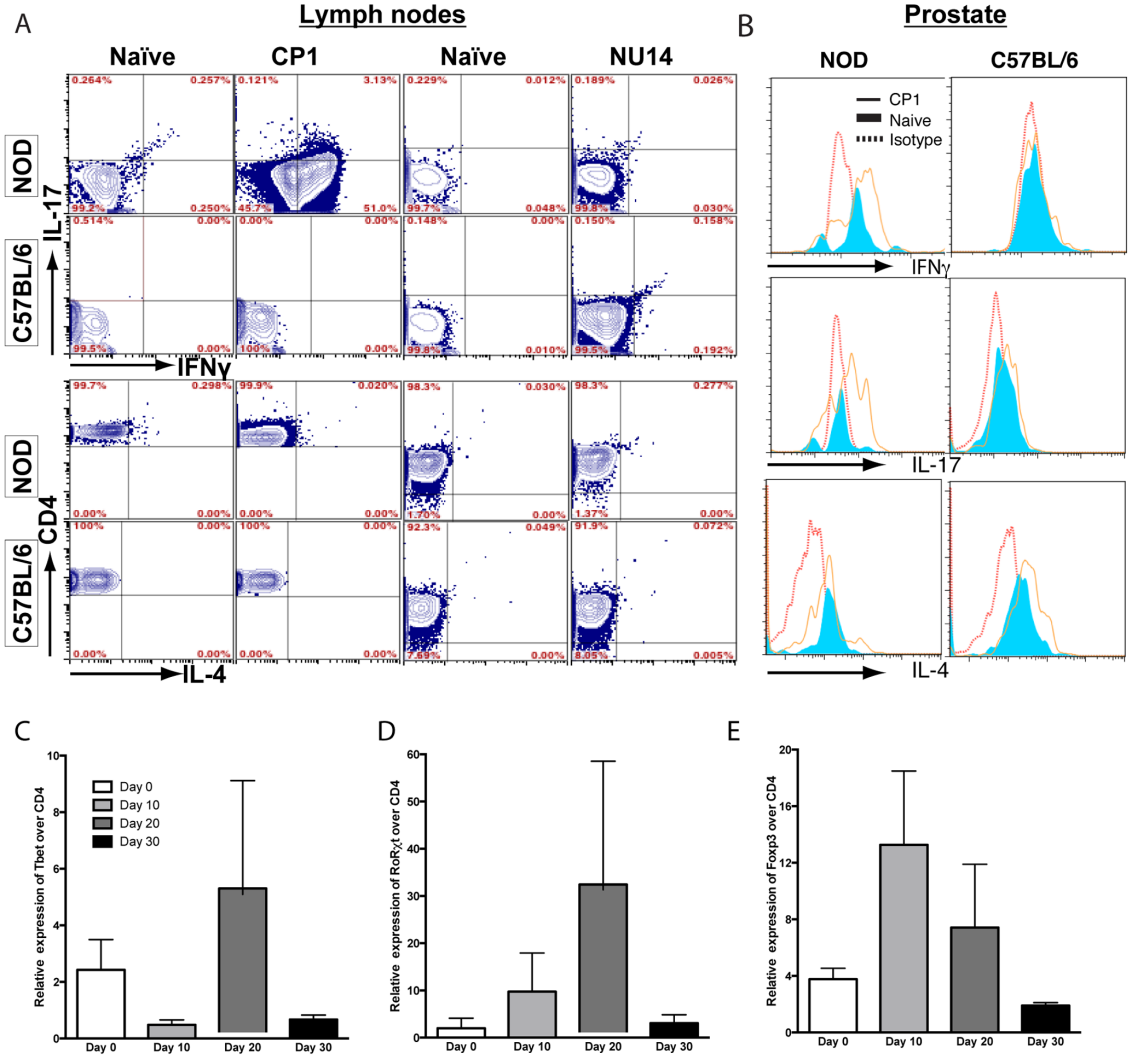
CD4+ T cell subsets in lymph nodes and prostates of CP-1-infected mice. (A) Representative flow cytometry analysis of CD4+ T cells from lymph nodes of naïve and CP-1 infected NOD and B6 mice. Isolated lymph node cells were gated on CD4+ T cells followed by IFN-γ+, IL-17A+, IFN-γ+ IL-17A+, and IL-4+ expression. (B) Representative flow cytometry analysis of intraprostatic CD4+ T cells from naïve or CP-1 infected NOD and B6 mice. Histograms representing intracellular cytokine staining in CP-1 infected (solid line) NOD or B6 mice were overlaid with naïve T cells (tinted) and isotype controls (dotted line). Each experiment was independently repeated at least three times with 5 mice/group. In real-time PCR assays, prostates from NOD mice infected with CP1 were used to prepare cDNA and real-time PCR was performed for lineage-specific transcription factors Tbet, Foxp3, RORγt, as well as CD4. Expression levels are shown as Ct(TF)/Ct(CD4) and is the average of three independent experiments.

In previous studies we have demonstrated that infection of the NOD prostate by CP-1 or the closely related UPEC cystitis isolate NU14 lead to different pelvic pain outcomes, with CP-1 alone capable of producing sustained pelvic pain [8]. We hypothesized that CD4+ T cells are differentially induced in a host and pathogen-specific fashion to drive responses that lead to chronic pelvic pain. We examined the functional phenotype of CD4+ T cells isolated from the lumbar and caudal lymph nodes of CP-1 infected mice at 28 days (five per group) using intracellular cytokine staining for IFN-γ, IL-4 and IL-17A as representative markers for Th1, Th2 and Th17 CD4+ T cells. CP-1 infected NOD mice demonstrated a significant increase in IFN-γ (p<0.05) producing CD4+ T cells, but no significant change in IL-17A, IFN-γ plus IL-17A or IL-4-expressing CD4+ T cells in the lymph nodes when compared with naïve controls (Fig. 4A). In contrast, NOD mice infected with the non-pain inducing UPEC strain NU14 (five per group) demonstrated no change from naïve controls with regard to IFN-γ, IL-17A or IL-4 (Fig. 4A). These results suggest that the CD4+ T cell immune response in lymph nodes to CP-1 infection in NOD mice is predominantly a Th1 response and appears to be tailored to the CP-1 pathogen. We next examined the immune response in B6 mice to CP-1 and NU14 infection (five per group). CP-1 infected B6 mice did not show the ability to mount a significant IFN-γ, IL-17A or IL-4 CD4+ T cell response (Fig. 4A). In response to NU14 infection in B6 mice, there was no significant IFN-γ, IL-17A or IL-4 response in the local lymph nodes. These results suggest that the CD4+ T cell response in local lymph nodes to prostate infection is dependent on the host strain as well as the strain of infecting microbe.

We next examined whether a local CD4+ T cell response to CP-1 infection was present in the prostate of NOD and B6 mice. Following enzymatic single cell dissociation, prostate cells were functionally phenotyped similarly to the lymph nodes. CP-1 exposed NOD mice (28 days, five per group) showed a significant increase in IFN-γ (p<0.05) and IL-17A (p<0.05) expression but no significant change from naïve controls with regard to IL-4 expression (Figure 4B). In B6 mice exposed to CP-1, we failed to observe a significant change from naïve controls with regard to IFN-γ, IL-17A or IL-4 expression. Due to the limited number of T cells observed in the prostate of CP1-infected mice, we further utilized quantitative PCR of the NOD prostate (five per group) to evaluate CD4+ T cell subsets in the prostate using relative levels of the transcription factors Tbet, RORγt and FoxP3 for Th1, Th17 and Treg subsets respectively (Figure 4C–E). Data demonstrates that all CD4+ T cell subsets were initially low. By Day 10, regulatory T cells have expanded, presumably to inhibit the acute immune response as a result of CP1 infection. Similarly, RORγt is also increased at Day 10, suggesting that Th17 cells are involved in the immune response. At day 20, in keeping with a diminished regulatory T cell response, both Th1 and Th17 transcription factors are maximally expressed, with a return to baseline levels by day 30 after infection with CP1. These results suggest an active Th1/Th17 response in the prostate that is under normal regulation by regulatory T cells.

### IFN-γ and IL-17A-expressing cells are sufficient to induce pelvic pain in NOD mice

**Figure 5:**
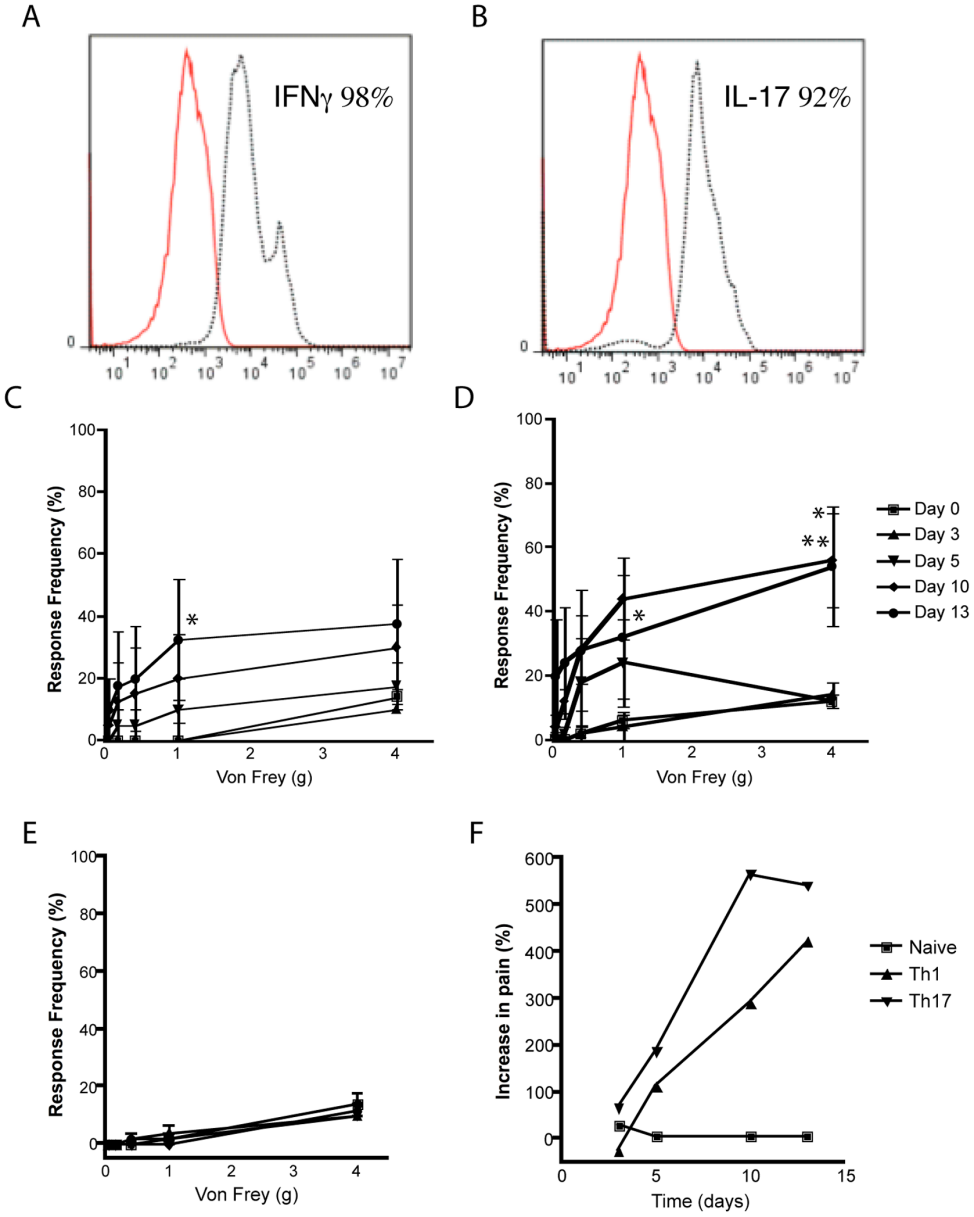
Adoptive transfer of IFNγ+ or IL-17A+ CD4+ T cells mediate pelvic pain. (A and B) T cells from CP-1 infected NOD mice were cultured under Th1 (A) or Th17 (B) polarizing conditions followed by flow cytometry using anti-IFN-γ and anti-IL-17A (dotted line) or isotype control (solid line) antibodies. (C-F) Naive NOD mice were injected with 1×10^6^ IFN-γ+ (C), IL-17A+ (D), or naïve T cells (E) and referred visceral hyperalgesia in NOD mice was measured using von Frey filaments of 5 calibrated forces. Data is shown as the mean percentage of positive response (+/−) SEM before transfer of T cells (day 0) and at days 3, 5, 10 and 13 after transfer (C–E). (F) Percent increase in response frequency from baseline in NOD mice injected with CD4+ T cells. Experiments were repeated twice independently and statistical significance is shown at *p<0.05 or **p<0.01.

We examined whether the IFN-γ and IL-17A expression was a consequence or a causative mechanism for pelvic pain in NOD mice. Cells from CP-1 infected mice were expanded *ex vivo* under Th1 and Th17 polarizing conditions, selected to greater than 90% purity and adoptively transferred into naïve NOD recipients (Fig. 5A and B, five per group). Both IFN-γ and IL-17A transferred mice exhibited a step-wise increase in tactile allodynia that was statistically significant by day 10 in IL-17A transfers (Fig. 5C and F) and by day 13 in IFN-γ transferred NOD mice (Fig. 5D and F). In contrast, adoptive transfer of naïve T cells did not result in an increase in response frequencies (Fig. 5E and F). These results indicate that IFN-γ and IL-17A producing T cells are sufficient by themselves to induce chronic pelvic pain.

### NOD-IFN-γ-KO mice exhibit enhanced pelvic pain and lymph node expression of IL-17A

**Figure 6:**
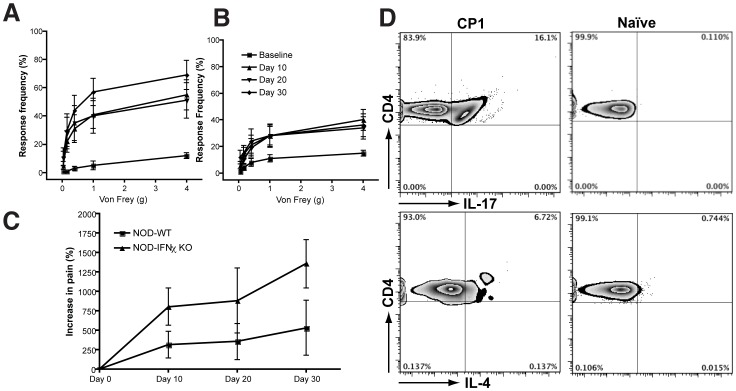
NOD-IFN-γ-KO mice exhibit enhanced pelvic pain and lymph node expression of IL-17A. NOD-IFN-γ-KO (A, n = 5) or NOD mice (B, n = 5) or were infected with CP-1 bacteria and referred visceral hyperalgesia was measured as responses to mechanical stimulation of the pelvic region using von Frey filaments of 5 calibrated forces. Data is shown as the mean percentage of positive response ± SEM before infection (baseline) and at days 10, 20 and 30 days after infection. The symbol key shown in panel B applies to panels A. Percent increase in response in NOD and NOD-IFN-γ-KO mice were compared over the 30 days time-course (C). Representative flow cytometry analysis of CD4+ T cells from draining lymph nodes of naïve and CP-1 infected NOD-IFN-γ-KO mice. Isolated lymph node cells were gated on CD4+ T cells followed by intracellular cytokine staining for IL-17A+ and IL-4+ expression. Percent of CD4+ T cells representative of multiple animals are shown in individual quadrants. Experiments were repeated twice independently with 5 mice/group.

We examined the immune response as well as the development of pelvic pain in NOD-IFN-γ-KO mice infected with CP1 and compared it to wild-type NOD mice. NOD-IFN-γ-KO mice (five per group) exhibited a stepwise increase in tactile allodynia that was significantly different from baseline (p<0.01) at 10, 20 and 30 days following CP1 infection (Fig. 6A). Wild-type NOD mice (five per group) also demonstrated a significant increase in tactile allodynia from baseline (p<0.01) at the same time-points (Fig. 6B) following CP1 infection. In comparing the increase in pain between the two strains following CP1 infection (Fig. 6C), there was a significant enhancement of the pelvic pain response in NOD-IFN-γ-KO mice compared to wild-type NOD mice (p = 0.0289). We also examined bacterial colonization of the prostates of these mice at day 30 with CP1 infection and did not observe any significant bacterial colonization in NOD-IFN-γ-KO mice similar to wild-type NOD mice (data not shown). These results suggest that the absence of IFN-γ does not appreciably affect the ability to clear bacteria from the prostate. We next examined the draining lymph nodes for expression of IL-17A and IL-4 using intracellular cytokine staining. CD4+ T cells from CP1 infected NOD-IFN-γ-KO mice showed enhanced production of IL-17A as well as a small increase in IL-4 when compared to naïve controls (Fig. 6D). These results contrast with our observations of a lack of IL-17A or IL-4 expression in draining lymph nodes of wild-type NOD mice (Fig. 4A) and suggest a compensatory increase in Th17/Th2 cytokine production in the absence of IFN-γ.

## Discussion

CP/CPPS has been postulated to be associated with microbial insult to the prostate but the evidence for this etiology has been hard to pinpoint with disease diagnosis occurring at a time-point discrete from the inciting event. In an earlier study, we presented the first experimental evidence that a bacterial isolate from a patient with CP/CPPS can initiate and sustain the development of chronic pelvic pain, a distinguishing characteristic of CP/CPPS [8]. However, beyond demonstration of an important role for the host genetic background and the virulence of the infecting microbe, the mechanism underlying chronic pelvic pain remained to be solved. In this study we demonstrate that chronic pelvic pain initiated though a microbial infection of the prostate is induced by a Th1/Th17 immune response that is closely tied to the nature of the offending pathogen and the genetic background of the infected host.

Studies from a number of laboratories, including ours, have demonstrated the ability of UPEC delivered transurethrally to infect the murine prostate and induce inflammation [8], [13]–[16]. In our previous study utilizing the CP1 bacterial isolate, the presence of inflammation five days after infection was observed in NOD and B6 mice and not observed to correlate with the development of chronic pelvic pain. Consistent with these earlier findings, chronic inflammation at 28 days, as measured by leukocytic infiltration, did not appear to be different between the two strains of mice (Fig. 1A). Examination of the chemokine profiles induced in the prostate revealed differential expression of a small number of cytokines/chemokines in NOD and B6 mice (Fig. 1D). In NOD mice, elevation of the CXC chemokine MIP-2 is suggestive of neutrophil activation and recruitment [17]. In B6 mice, elevated expression of IL-16, a pro-inflammatory cytokine chemotactic for CD4+ T lymphocytes, monocytes, and eosinophils and IL-1ra, a receptor antagonist of IL-1 cytokines was observed. The increased size of secondary lymphoid organs in the NOD mouse is suggestive of robust immune activation in NOD mice.

Chronic prostatitis in humans is associated with the presence of specific auto-antibodies [18] as well as auto-reactive T cells [19]–[22]. In this study, we demonstrate that adoptive transfer of T cells and more specifically CD4+ T cells from CP-1 infected donors is sufficient to mediate the development of pelvic pain. Our results are consistent with a reported requirement for CD4+ T cells in the NOD autoimmune prostatitis model [23] as well as a well-studied requirement for CD4+ T cells in the pathogenesis of type-1 diabetes in the NOD genetic background [24]. CD8+ T cells, however, displayed an ability to mediate pelvic pain irrespective of whether they were derived from naïve or CP1-infected NOD mice. These nonspecific results suggest that endogenous, antigen-specific CD8+ T cells in naïve NOD mice are capable of undergoing activation without co-stimulation [25], [26] and expanding in an non-specific antigen-independent manner [27] to mediate pelvic pain. We have previously demonstrated that infection of the NOD prostate by CP1 or the closely related UPEC cystitis isolate NU14 lead to different pelvic pain outcomes, with CP1 alone producing sustained chronic pelvic pain [8]. In this study, we show that lymph node derived CD4+ T cells from CP1 infection are characterized by significant intracellular expression of IFN-γ, a signature cytokine for Th1 helper T cells. NU14 by contrast was incapable of such a response indicating the pathogen specificity of the immune response. The absence of IL-4, signifying a lack of a Th2 response to CP1 is consistent with the inability of immune serum to transfer chronic pelvic pain. Our results agree with previous studies demonstrating an important role for IFN-γ in the development of autoimmune prostatitis in the NOD mouse [28], but represents, to the best of our knowledge, the first demonstration of a Th1 response that leads to chronic pelvic pain. In contrast to the immune response in the local lymph node, infiltrating CD4+ T lymphocytes in the NOD prostate were characterized by increased IFN-γ and IL-17A expression in response to CP1 infection. IL-17A has been reported to be elicited from γδ T cells in UPEC bladder infections in a murine model and appear to be primarily functional at the level of innate immunity [29]. In contrast, our results demonstrate expression of IL-17A in αβ CD4+ T cells in a host and pathogen specific manner. Expectedly, the increase in IFN-γ and IL-17A transcription factors is associated with a diminished regulatory T cell response.

Beyond mere association with chronic pain responses, both IL-17A and IFN-γ appear to be sufficient to mediate chronic pelvic pain. IL-17A is a potent regulator of matrix metalloproteases, acute phase proteins, IL-6, and CXC chemokines that function to recruit neutrophils leading to their mobilization, recruitment, and activation. IL-17A may mediate hyperalgesia and pronociceptive effects through neutrophils [30] or alternatively through direct mechanisms that contribute to sensitization and allodynia [31]–[33]. In this context, it is worth noting that IL-17A has been demonstrated to be an upstream mediator of CCL2/MCP-1 expression [34]. CCL2/MCP-1 is elevated in prostate secretions of men with CP/CPPS and is a potential biomarker with a role in CP/CPPS pathogenesis [35]. IFN-γ, a well-known pro-inflammatory cytokine has also been implicated in hypernociception [36] and neuropathic hypersensitivity through T cell activation induced mechanisms [37]. Our studies show an exacerbated pain response in NOD-IFN-γ-KO mice when compared to NOD mice infected with CP1. Furthermore, while IL-17 was not expressed in the draining lymph nodes of wild type NOD mice, IL-17 is greatly expressed in the lymph nodes of IFN-γ-KO CP-1-infected mice. These observations while counterintuitive, are supported by studies in autoimmune disease models such as experimental autoimmune encephalitis (EAE) where, in the absence of IFN-γ, disease progression in EAE is exacerbated and IFN-γ is believed to act as an inhibitor of disease initiation [38]–[41]. The key cytokine responsible for the proinflammatory autoimmunity in these models appear to be IL-17A [38] and IFN-γ is known to inhibit Th17 polarization. Thus, in its absence, Th17 development and IL-17A expression is expected to progress unimpeded and promote disease/inflammation. Taken together, it is not surprising that the increase in pelvic pain observed in the NOD-IFN-γ-KO mice is associated with an increase in IL-17A.

In summary, we show that a Th1 and Th17 immune response is responsible for pain in CP-1-infected NOD mice while C57BL/6 mice infected with CP-1 and NOD mice infected with NU14 fail to mount a Th1/Th17 immune response. Our finding that antigen-experienced Th1 and Th17 cells transfer pain into naïve mice demonstrates that inflammatory T cells are the main mediators of pain in the mouse infection model of CP/CPPS. Important questions that remain to be answered and a future focus of our studies is identifying the effector cells/mechanisms activated by IL-17A and IFN-γ in the prostate that mediate disease pathology and symptoms. In the context of human CP/CPPS, our results strongly argue for systematic examination of the immune profile of patients with chronic pain to identify subsets of individuals that may have active Th1/Th17 mechanisms. Thus, these results have potentially important implications in understanding the pathogenesis of CP/CPPS.

## Materials and Methods

### Bacterial strains and cell lines

CP-1 is an *E. coli* strain of the B1 phylogenetic group from the expressed prostatic secretion and post-massage voided urine of a patient with CP/CPPS [42]. NU14 is an *E. coli* strain of the B2 phylogenetic group isolated from the urine of a patient with acute cystitis [43]. Bacteria for *in vivo* infections were prepared as previously described [44]. The prostate stromal cell line BPH-1 was a kind gift from Dr. Chung Lee (Northwestern University, Chicago, IL) [45]. PEC-1 epithelial cells were derived from a healthy adult prostate and immortalized by introduction of human papillomavirus type 16 E6E7 [8]. Prostate antigens for in vitro assays were prepared by intermittent sonication [46] (amplitude of 70% for one minute, pulsed 10 s with 20 s intervals) over ice or by extraction in Triton X100.

### Animal infection

NOD/ShiLtJ (NOD) and C57BL/6 (B6) (5 to 7 weeks old) mice were purchased from Jackson Laboratory (Bar Harbor, ME). NOD.129S7(B6)-Ifngtm1Ts/DvsJ (NOD-IFN-γ-KO) mice were a kind gift from Dr. David Serreze at Jackson Laboratory (Bar Harbor, ME) and were used for breeding at the Center for Comparative Medicine, Northwestern University. To infect animals, 10 µl of phosphate-buffered saline containing 1×10^8^ bacteria was introduced into the urethra of anesthetized male mice by catheterization [8]. Bacterial colonization of the prostates were assessed as previously described [8].

#### Ethics statement

All experiments were approved by the Northwestern University Animal Care and Use Committee.

### Behavior testing

Behavior testing was based on the concept of cutaneous hyperalgesia resulting from referred visceral pain [10]–[12]. An irritable focus in visceral tissues reduces cutaneous pain thresholds allowing for an exaggerated response to normally non-painful stimuli (allodynia). Mice were tested for allodynia in individual Plexiglas chambers (6×10×12 cm) with a stainless steel wire grid floor as previously described (Fig. S1) [46]. Referred hyperalgesia and tactile allodynia were tested using von Frey filaments with forces of .04, .16, .4, 1 and 4 g (Stoelting) (1). Each filament was applied for 1–2 s with an inter-stimulus interval of 5 s for a total 10 times, and the hairs were tested in ascending order of force. Stimulation was confined to the lower abdominal area in the general vicinity of the prostate and care was taken to stimulate different areas within this region to avoid desensitization or “wind up” effects. An investigator blinded to the group treated graded responses in animals. Three types of behaviors were graded as positive responses to filament stimulation: *1)* sharp retraction of the abdomen; *2)* immediate licking or scratching of the area of filament stimulation; or *3)* jumping. Response frequency was calculated as the percentage of positive response (out of 10, e.g., 5 responses of 10 = 50%), and data were reported as the mean percentage of response frequency ± SEM [47].

### Chemokine antibody assays

Prostate lysates from mice that were uninfected or infected with CP1 for 30 days (n = 5/group) were equalized for protein concentration and used in a chemokine antibody array as per manufacturer's protocol (ARY006, R&D Systems). Briefly, lysates were mixed with biotinylated cytokine specific detection antibodies (R & D systems) followed by incubation with array membranes that bound antibody complexes. Following washes, chemiluminescence was used to detect bound cytokine.

### Cell Separation and transfer

#### (i) Prostate cells

For experiments involving single-cell suspensions of the prostate, enzymatic methods were utilized for isolation. Prostates were rinsed with complete RPMI 1640 containing 10% FBS, cut into small pieces and digested for 40 minutes at 37°C with 1 mg/mL collagenase D (Roche Diagnostics), 10 mM HEPES (Mediatech) and .01% DNase I (Sigma) in complete RPMI 1640 (Mediatech). Digested suspensions were passed through a 40 µm nylon mesh and centrifuged. The cell pellet was re-suspended in FACS staining buffer followed by analysis and gating on CD4+ T cells (Fig. S2).

#### (ii): T cells

Pan T Cell, CD8+ T Cell and CD4+ T Cell isolation kits (Miltenyi Biotec) were used to isolate cells using negative depletion as per manufacturers protocol. Pan T, CD4+ T, CD8+ T cells and serum were derived from naïve mice or mice that had been infected with CP-1 for 28 days. 4×10^6^ T cells or 100 µL of serum were injected into recipient mice intravenously during immune transfer [48].

### *Ex vivo* T cell skewing

On day 30, CP-1 infected and uninfected NOD mice (controls) were euthanized and lumbar lymph nodes were isolated and made into single cell suspension. Cells were washed twice with complete 10% RPMI media. 1–2×10^6^ cells were plated in wells containing plate bound anti-CD3/CD28 and either Th1 (5 µg/ml anti-IL-4 and 1 ng/ml IL-12) or Th17 (2 µg/ml IL-6, 20 µg/ml IL-23, 3 µg/ml TGF-β, 5 µg/ml anti-IL-4, 5 µg/ml anti-IFNγ) polarizing cytokines for 5 days. On the fifth day, IFN- γ and IL-17A secreting cells were isolated using the mouse IFN-γ and IL-17A secretion assay kits (Miltenyi Biotec) according to manufacturer's protocol and adoptively transferred intravenously into naïve NOD mice.

### Analysis of CD4+ T cell subsets

Phenotyping of CD4+ T cells for intracellular IFN-γ, IL-17A and IL-4 expression was performed using the mouse Th1/Th2/Th17 phenotyping kit (BD Pharmingen) following manufacturer's protocol. Briefly, after cell separation, lymphocytes and prostate cells were resuspended in cold BD Cytofix Buffer for 20 minutes followed by permeabilization and incubation with anti-CD4+ PerCP-Cy5.5, anti-IL17A-PE, anti-IFN-γ-FITC and anti-IL-4 APC. Stained cells were analyzed on an Accuri C6 flow cytometer and analysis of cell populations was performed using FlowJo (Treestar) software. For real-time PCR assays prostates were isolated, dissociated into single cell suspensions, and stimulated with ionomycin and PMA for 4 hours. After 4 hours, cells were lysed and mRNA was isolated using the SV Total RNA Isolation System (Promega). cDNA was reverse transcribed (SuperScript III Cells Direct cDNA Synthesis Kit, Invitrogen) from mRNA and RT-PCR was performed for lineage-specific transcription factors Tbet, Foxp3, RORγt, as well as CD4. Expression levels are shown as Ct(TF)/Ct(CD4) and is representative of three separate experiments.

### Proliferation assay

The ability of T cells from 28 day CP1 infected NOD mice (N = 3 pooled) to undergo proliferation in an antigen specific manner was examined. Naïve NOD splenocytes were used as antigen presenting cells (APC) and incubated overnight following irradiation (3000 rad) with antigens (2 µg/mL); rat prostate lysates, mouse prostate lysates, antigens from human prostate epithelial cells (PEC-1), and human stromal sells (BPH-1). CD4+ T lymphocytes from CP1 infected NOD spleens (95% pure as determined by FACS analysis) were added to the antigen-pulsed splenocytes and incubated at 37°C for 3 days. At day 3 following culture, 150 µl of cell suspension was transferred into FACS tubes, stained with propidium iodide for viability and a set volume of 100 µl was used to perform cell counts. Absolute cell counts were obtained using a BD Accuri® C6 flow cytometer from the viable cell population using previously established gates for T cells. All experiments were performed in triplicate and events per microliter was averaged. Results were expressed as fold change from day-1 counts and groups were compared using t-tests for statistical significance.

### Statistical analyses

Results were expressed as mean ± SEM and analyzed for statistical significance by unpaired *t* tests or ANOVA using Graphpad statistical software. A value of p<0.05 was considered statistically significant.

## Supporting Information

Figure S1 **Methodology for testing pelvic pain behavior using Von Frey fibers.** Mice were tested before prior to bacteria infrection and at postinfection days (PIDs) 1, 7, 14, 21, and 28. Three different types of behavior were considered as positive responses to filament stimulation: 1) sharp retraction of the abdomen; 2) immediate licking or scratching of the area of filament stimulation; or 3) jumping. Response frequently was calculated as the percentage of positive response (out of 10), and data were reported as the mean percentage of response frequency ± SE.
(PDF)

Figure S2 **Gating strategy for prostate-derived CD4+ T cells.** (A) The live prostate cell population was determined by excluding the propidium iodide (PI) stained cells from the overall cell population. Comparing the total lymphocyte prostate cell population (panel A, top left) (P3) to the live cell lymphocyte gate (panel A, top right) shows the percentage of lymphocytes is unchanged. (B) Representative flow cytometry analysis of CD4+ T cells from CP1 infected NOD prostate cells. Leukocyte populations in total prostate cells were identified using CD45.2 staining and used to establish the leukocyte gate followed by identification of CD4+ T cells.
(PDF)
