# Supplementary figures and images for: Th1-Th17 Cells Contribute to the Development of Uropathogenic Escherichia coli-Induced Chronic Pelvic Pain
Source: PLoS One. 2013 Apr 5;8(4):e60987. doi: 10.1371/journal.pone.0060987 (PMC3618515; doi:10.1371/journal.pone.0060987)

# Figure S1

Methodology for testing pelvic pain behavior using Von Frey fibers

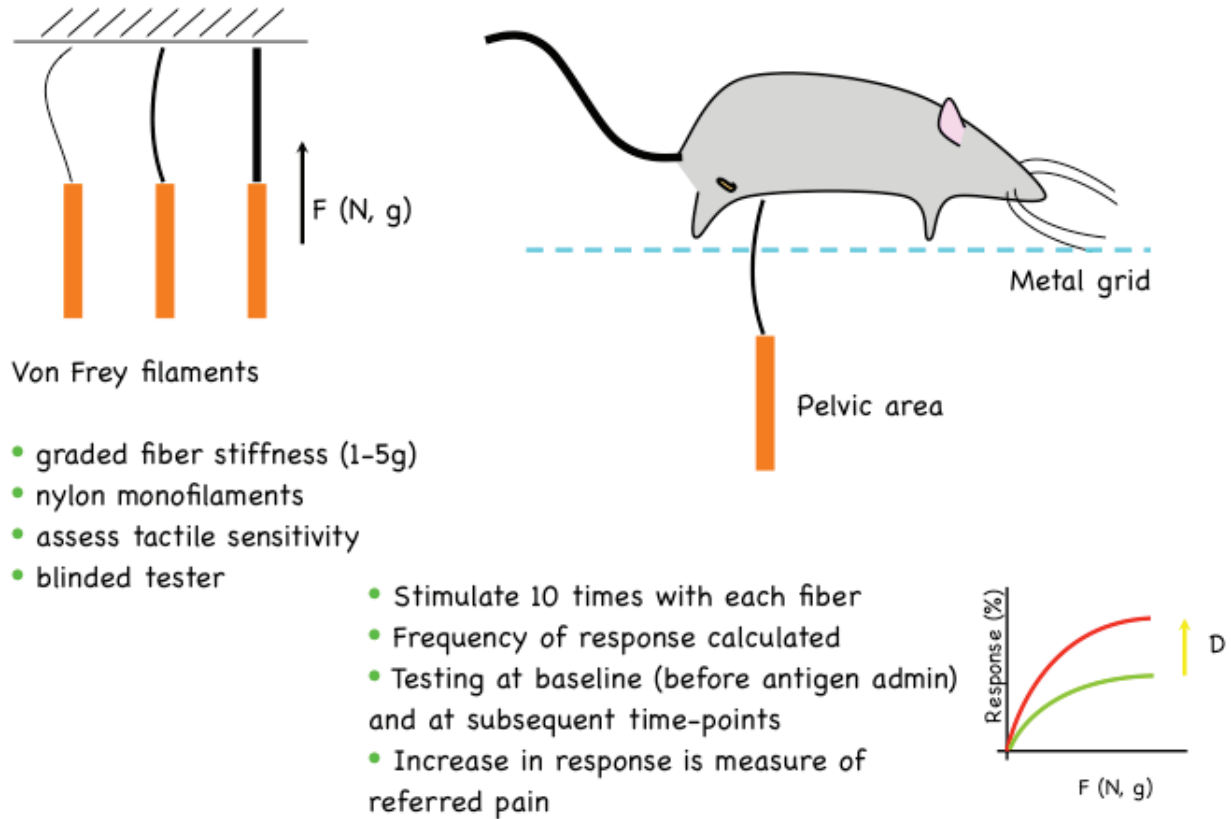

Supplement: Figure S1 — Methodology for testing pelvic pain behavior using Von Frey fibers. Mice were tested before prior to bacteria infrection and at postinfection days (PIDs) 1, 7, 14, 21, and 28. Three different types of behavior were considered as positive responses to filament stimulation: 1) sharp retraction of the abdomen; 2) immediate licking or scratching of the area of filament stimulation; or 3) jumping. Response frequently was calculated as the percentage of positive response (out of 10), and data were reported as the mean percentage of response frequency ± SE. (PDF) [file pone.0060987.s001.pdf]

**A**

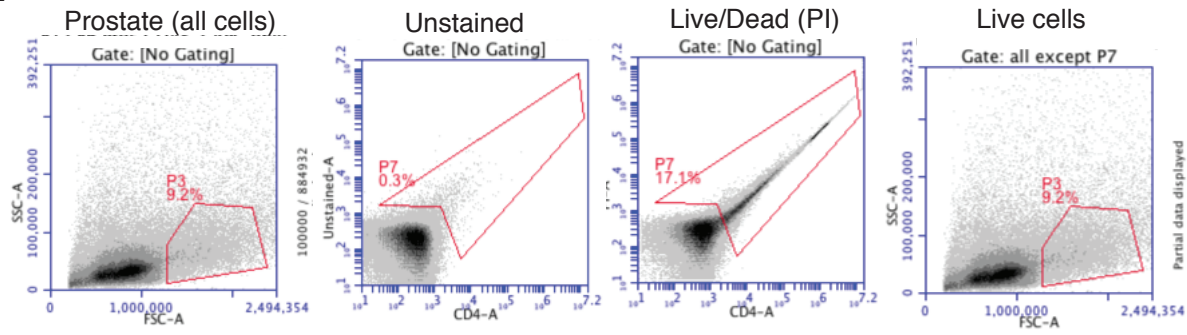

**B**

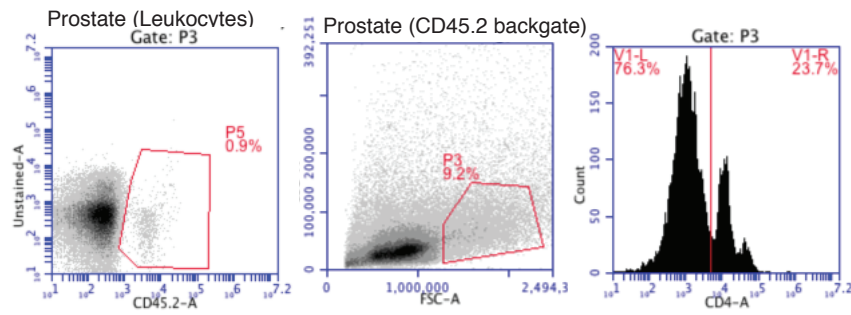

Supplement: Figure S2 — Gating strategy for prostate-derived CD4+ T cells. (A) The live prostate cell population was determined by excluding the propidium iodide (PI) stained cells from the overall cell population. Comparing the total lymphocyte prostate cell population (panel A, top left) (P3) to the live cell lymphocyte gate (panel A, top right) shows the percentage of lymphocytes is unchanged. (B) Representative flow cytometry analysis of CD4+ T cells from CP1 infected NOD prostate cells. Leukocyte populations in total prostate cells were identified using CD45.2 staining and used to establish the leukocyte gate followed by identification of CD4+ T cells. (PDF) [file pone.0060987.s002.pdf]
